# Supplementary material for: OPG/TRAIL ratio as a predictive biomarker of mortality in patients with type A acute aortic dissection
Source: Nat Commun. 2021 Jun 7;12:3401. doi: 10.1038/s41467-021-23787-5 (PMC8185077; doi:10.1038/s41467-021-23787-5)
Supplement: Supplementary file 1 — Supplementary Information [file 41467_2021_23787_MOESM1_ESM.pdf]

Supplementary Information

OPG/TRAIL ratio as a novel biomarker of mortality in patients with type A acute aortic dissection

Jie Lu, Ping Li, et, al.

CONTENTS

Supplementary Methods……………………………………………………………………………3
Supplementary Table 1………………………………………………………………………......9
Supplementary Table 2……………………………………………………………………….....10
Supplementary Table 3……………………………………………………………………….....12
Supplementary Table 4……………………………………………………………………….....14
Supplementary Table 5……………………………………………………………………….....15
Supplementary Table 6……………………………………………………………………….....16
Supplementary Table 7……………………………………………………………………….....17
Supplementary Table 8……………………………………………………………………….....18
Supplementary Table 9……………………………………………………………………….....19
Supplementary Figure 1…………………………………………………………………………20
Supplementary Figure 2………………………………………………………………………..21
Supplementary Figure 3…………………………………………………………………………22
Supplementary Figure 4…………………………………………………………………………24
Supplementary Figure 5…………………………………………………………………………25
Supplementary Figure 6……………………………………………………………………….....26
Supplementary Figure 7……………………………………………………………………….....27
Supplementary Figure 8……………………………………………………………………….....28

Supplementary Methods

Sample Size Calculation

Calculation of sample size were considered in different stages. In derivation cohort, to achieve 90% power at 5% significance with a 4:1 sampling ratio (because 25% of reported mortality rate), we required 504 participants to detect an overall survival hazard ratio of 1.5. In validation cohort, the sample size estimation based on preliminary data of derivation cohort. Assuming a mortality rate of 20%, a sample size of 187 patients would be powered (90% at $p<0.05$) to detect a HR of OPG/TRAIL ratio of 2.0.

Type A acute aortic dissection (TA-AAD)–associated definitions

Three independent physicians evaluated the medical charts reporting all triage data, such as presenting symptoms, detailed medical history, and physical examination results.

1) Type and acute definitions: Dissection type was determined according to Stanford classification: it was defined as acute if the diagnosis was made within 14 days of symptom onset.

2) Clinical characteristics at admission: malperfusion syndrome was defined as signs or symptoms attributable to disrupted blood flow to an end organ system, including cerebral, nervous, spinal, coronary, visceral, or renal systems and extremities. False lumen was defined as thrombosed when thrombus was partially or completely present and as non-thrombosed when flow was present without evidence of thrombus at any level of the aorta. A state of shock was defined as having systolic blood pressure (SBP) at admission less than 90 mm Hg, cardiac index less than 2.0 L/min/m^2, or a need for intravenous inotropic agents. Management was defined as surgical treatment when patients underwent operative repair or endovascular treatment. Pain features noted were migrating, abrupt onset, tearing, and intensive. Pain severity was defined by explicit definition of severity by the attending physician on the chart or by the administration of any opioid drug or analgesic drugs. Coma was diagnosed by a neurology consultant. The cumulative range of dissection was quantified and presented on a scale from 1 to 6 on the basis of involvement of the following segments: ascending aorta, aortic arch, descending aorta, supra-renal abdominal arteries, infrarenal abdominal arteries, and iliac arteries. Myocardial infarction (MI) was defined as a patient having a cardiac troponin I level above the 99th percentile, with at least 1 of the following: diagnostic serial electrocardiographic changes consisting of new pathological Q waves or ST-segment and T-wave change.

3) Surgical management refers to patients who received open surgical intervention.

Blood sample collection

A peripheral venous blood sample was drawn from all patients within 48 hours of admission before administration of surgical treatment and in a fasting state. Blood samples were drawn into coagulation-promoting tubes and centrifuged at 2000 *g* for 10 min within 1 h of collection. All serum or plasma samples were stored in aliquots at −80°C.

Antibody arrays

On the basis of budget limitations and time availability, we initially performed antibody arrays on samples from 12 patients with TA-AAD and 12 healthy controls (HC). The relative concentrations of soluble proteins in the serum were measured by using Human Antibody Array 1000 (a combination of Human L-507 and Human L-493, RayBiotech Inc.) according to the manufacturer's instructions. Briefly, each serum sample was hybridized to the arrays overnight at 4°C. All slides were scanned by using a GenePix 4000B Microarray Scanner and analyzed by using GenePix Pro 6.0 software. The results were then normalized by using internal controls. All results are available in Supplementary Table 2. We measured the relative concentrations of 26 soluble proteins in the serum from 31 patients with TA-AAD and 32 HC by using a custom antibody array ordered from RayBiotech company according to the same method described above.

Filtering pipeline to find candidate proteins

Forty-one candidate proteins were searched on PubMed, and the citation counts for aortic dissection-related pathological processes (including smooth muscle cell [SMC] proliferation, inflammation, acute stress response, extracellular matrix synthesis and degradation, angiogenesis, SMC apoptosis and thrombosis, and fibrinolysis) were recorded. We used log (counts+1) as the metric, defined as PubMed Rank Score, for sorting the relevance of proteins to the aortic dissection-related pathological process. Meanwhile, we applied Elasticsearch based on Lucene (https://github.com/elastic/elasticsearch) on the sorting of protein relevance with aortic dissection-related pathological processes. The sum of matching score (ES Scores) for each protein among all AAD-related pathways is defined as log (ES Scores+1). Finally, we used a summarized metric (defined as log (counts+1) ×log (ES Scores+1)), integrating both ranking methods, to find the proteins most related to aortic dissection-related pathological processes. The larger the summarized Rank Score, the more relevance the protein has to aortic dissection-related pathways. As a result, 26

target proteins were selected as final candidates related to aortic dissection (Supplementary Fig. 2). Among the 26 candidate proteins, the top-ranking protein was D-dimer (well-known biomarker of aortic dissection), suggesting that our selection method is effective (Supplementary Fig. 2a).

Enzyme-linked immunosorbent assay (ELISA)

Levels of 13 circulating proteins were measured via standard enzyme-linked immunosorbent assay (ELISA) kits (Ray Bio, Norcross, GA, USA): plasminogen (PLG), lipocalin (LCN)-2, serum amyloid A (SAA)1, fibronectin (FN)-1, vitronectin (VTN), angiopoietin-1(ANGPT1), platelet factor 4 (PF4), transforming growth factor β receptor（TGF-βR）-III）, osteoprotegerin (OPG), ciliary neurotrophic factor (CNTF), fibroblast growth factor (FGF)-21, vascular endothelial growth factor (VEGF)-B, and oxidized low-density lipoprotein receptor (LOX)-1. TNF-related apoptosis-inducing ligand (TRAIL) levels were determined by performing an available ELISA (R&D Systems, Minneapolis, MN, USA). All measurements were taken in duplicate. If the detectable value was below the detection limit, then the value was replaced with the detection limit or the value of sensitivity. The respective inter- and intra-assay coefficients of variation in TA-AAD patients were as follows: LOX1, 3.92% and 4.37%; TRAIL, 3.87% and 4.32%; LCN2, 3.31% and 4.60%; OPG, 3.41% and 3.80%; FN1, 4.61% and 3.68%; PLG, 3.79% and 3.74%; ANGPT1, 2.71% and 3.33%; PF4, 3.52% and 4.27%; and SAA1, 4.11% and 3.96%.

Study Protocol

Brief Summary:

Type A acute aortic dissection (TA-AAD) is a catastrophic condition caused by dissection of the ascending aorta. Despite many therapeutic improvements, short-term and long-term mortality risk remain high. Accurate risk stratification contributes to treatment decision-making and improvement of the prognosis. However, little is known about specific biomarkers associated with prognosis. The study aims to identify a novel biomarker associated with death risk in patients with TA-AAD.

Detailed Description:

Aim 1: To identify candidate proteins closely related to TA-AAD, an unbiased screening process will be performed in the cross-sectional study.

Aim 2: To identify the novel biomarkers associated with the mortality risk in patients with TA-AAD, a multicentre, observational, prospective study will be performed at five hospitals in China. The prognostic value of candidate TA-AAD-specific proteins will be prospectively evaluated in

multicenter cohorts.

Aim 3: To develop a biomarker-based risk stratification tool, after selecting the prognostic proteins, a biomarker-based risk stratification will be derived and further verified in an independent cohort.

Study Design:

Study Type: Observational

Estimated Enrollment: 691 participants

Observational Model: Cohort

Time Perspective: Prospective

Official title: A Registry Study on Genetics and Biomarkers of Thoracic Aortic Aneurysm/Dissection (ARSGBTAAD)

Study Start Date: September 2014

Estimated Primary Completion Date: December 2019

Groups and Cohort:

Patients with Type A Aortic Dissection (TA-AAD) and Health controls

Outcome measures:

1. Preliminary assessment of candidate proteins related with type A acute aortic dissection (TA-AAD) [Time Frame: Baseline (prior to surgical treatment)]

Apply antibody arrays together with bioinformatics to screen candidate proteins related with AAD pathological process, and enzyme-linked immunosorbent assay (ELISA) to verify the levels of candidate proteins in TA-AAD patients and health control.

2. Correlation of candidate proteins with the long-term mortality risk in patients with TA-AAD [Time Frame: At least 12 months]

Define the primary endpoint as long-term mortality. Long-term mortality is defined as all-cause mortality following initial hospital admission until to death or follow-up endpoint. The serum concentrations of candidate proteins are tested in patients with TA-AAD by ELISA. Cox proportional hazards regression analysis is conducted to identify associations of candidate proteins with long-term mortality. Known risk factors (age≥70 years, systolic blood pressure, and smoking), reported potential predictors (pain, malperfusion, shock or hypotension, coma or stroke), and imaging indicators (thrombosis in a false lumen and segments), and surgical treatment are considered as covariates. The discrimination and reclassification are analyzed by Harreller's C-

index and NRI.

3. Correlation of candidate proteins with risk of short-term mortality in patients with TA-AAD [Time Frame: after admission]

Define the secondary endpoints as short-term mortality, including inhospital or 30-day mortality. Discharged day or day 30 after admission are set as the start of the follow-up period.

4. Comparison of predictive performance between biomarkers and the existing clinical models or predictors [Time Frame: At least 12 months]

Harreller's C-index and NRI are calculated to estimate the improvement of candidate biomarkers compared to existing prediction model or clinical predictors. The C-index and NRI are tested using 1000 bootstrap resamples.

5. Development of biomarker–guided risk stratification tool in a derivation cohort [Time Frame: At least 12 months]

Receiver-operating characteristic curve analysis is performed to determine the cut-off value of the biomarker that predicted the long-term death risk. Cumulative death-free survival curves are derived using the Kaplan-Meier method and group-wise comparisons are based on the log-rank tests.

6. validation of biomarker–guided risk stratification tool in a validation cohort [Time Frame: At least 12 months]

The performance of biomarker-based risk stratification is validated in an independent cohort. According to the cut-off values of biomarkers derived from derivation cohort, we calculate its sensitivity, specificity, positive predictive value, and negative predictive value.

7. Correlation between candidate biomarkers and the risk of long-term death in different subgroups [Time Frame: At least 12 months]

Examine the association between biomarker and death in different subgroups (eg. male vs. female, smoking vs. non-smoking, hypertension vs. non-hypertension et al.).

Biospecimen Retention:

Peripheral venous blood samples are drawn from all patients before administration of surgical treatment. Blood samples are drawn into coagulation-promoting tubes and centrifuged at 2000 g for 10 min within 1 h of collection. All serum samples are stored in aliquots at −80°C.

Eligibility Criteria:

Ages Eligible for Study: up to 85 years old

Sexes Eligible for Study: All

Accepts Healthy Volunteers: Yes

Sampling Method: Non-probability Sample

Study Population: TA-AAD Patients

Criteria:

Inclusion Criteria:

Patients diagnosed as TA-AAD are involved. The diagnosis of TA-AAD was confirmed based on patient history and imaging tests (computed tomography angiography or transthoracic echocardiography). The acute phase of aortic dissection was defined as within 2 weeks from symptom onset to hospital admission.

Exclusion Criteria: Patients with congenital heart disease, recent surgery, malignancy, systemic inflammatory disease, pregnancy, acute infective disease, no subsequent surgical repair, no signed informed consent, or severe lack of baseline data were excluded from the study.

Collaborators:

Beijing Anzhen Hospital of Capital Medical University and Beijing Institute of Heart Lung and Blood Vessel Diseases;

Xi Jing Hospital of Fourth Military Medical University;

First Affiliated Hospital of Sun yat-sen university;

The First Affiliated Hospital of Dalian Medical University;

Union Hospital Affiliated with Tongji Medical College, Huazhong University of Science and Technology

Principal Investigator: Dr. Yulin Li, Beijing Anzhen Hospital of Capital Medical University and Beijing Institute of Heart Lung and Blood Vessel Diseases;

Supplementary Table 1. Baseline characteristics of patients in three screening sets.

	Screening set 1			Screening set 2			Screening set 3		
	HC(n=12)	TA-AAD(n=12)	*p* value	HC(n=32)	TA-AAD(n=31)	*p* value	HC(n=200)	TA-AAD(n=77)	*p* value
Age, y	48.8±9.7	48.3±10.5	0.891	48.5±7.6	49.3±10.5	0.723	48.2±6.7	47.4±11.2	0.573
Men, n (%)	9(75.0)	9 (75.0)	1.000	25 (78.1)	24(77.4)	1.000	129(64.5)	56 (72.7)	0.193
BMI, kg/m^2	24.4±2.2	24.9±3.6	0.694	23.8±1.6	24.5±2.6	0.246	23.6±2.0	22.9±7.7	0.407
SBP, mmHg	128.7±13.2	134.5±9.3	0.221	122.8±11.9	134.0±12.2	4.740e-4	124.8±15.3	131.1±18.0	0.004
DBP, mmHg	79.6±9.0	81.5±8.7	0.520	80.0±7.5	78.7±9.6	0.562	81.6±14.7	79.4±9.8	0.140
Current smoking	5(41.7)	6 (50.0)	0.723	15 (46.9)	14(45.2)	1.000	89(44.5)	31 (40.3)	0.523
Hypertension, n (%)	9(75.0)	10 (83.3)	0.586	19 (59.4)	20(64.5)	0.797	121(60.5)	49 (63.6)	0.631
CAD history, n (%)	0(0)	1 (8.3)	1.000	0(0)	1(3.2)	0.492	10(5.0)	5 (6.5)	0.569
DM history, n (%)	2(16.7)	2 (16.7)	1.000	6(18.8)	6(19.4)	1.000	20(10.0)	5 (6.5)	0.362

Values are expressed as mean±SD, n (%), or median (interquartile range) unless otherwise indicated. HC=healthy control; TA-AAD=Type A acute aortic dissection; BMI= body mass index; SBP=systolic blood pressure, DBP=diastolic blood pressure, DM=diabetes mellitus, CAD=coronary artery disease. *p* values are two-tailed from paired *t*-tests in stage 1 or two-sample *t*-tests in stages 2 and 3 for continuous variables, and from chi-square tests for categorical variables.

Supplementary Table 2. Proteins that significantly differed between patients with TA-AAD and healthy controls in a 1000-antibody array.

Proteins abbreviation	Full name of proteins	A/H Fold change	*p* value
ACVR2A	Activin receptor type-2A	-1.67	4.252e-4
ADAM-9*	Disintegrin and metalloproteinase domain-containing protein 9	2.06	6.075e-6
ADAMTS-L2	Disintegrin and metalloproteinase with thrombospondin motif-like protein 2	-1.77	0.035
ALCAM*	Activated leukocyte cell adhesion molecule	-2.28	9.812e-6
ANG*	Angiogenin	-1.89	2.512e-5
ANGPTL3	Angiopoietin like-factor	-1.50	0.020
ANGPT1*	Angiopoietin-1	2.60	1.414e-6
ApoC2	Apolipoprotein C2	1.64	1.632e-6
CD40LG *	CD40 Ligand	-1.71	9.821e-6
CNTF*	Ciliary neurotrophic factor	1.66	9.498e-6
CST3*	Cystatin C	-1.69	8.503e-6
DCN*	Decorin	-2.71	7.489e-6
D-Dimer*	D-dimer	3.27	9.947e-6
EDA2R	Ectodysplasin A2 Receptor	-1.55	0.036
EphB6	EPH receptor B6	2.07	6.959e-6
EREG	Epiregulin	-1.61	9.884e-6
FAS/TNFRSF6*	TNF Receptor Superfamily Member 6	-1.80	9.749e-6
FGF21*	Fibroblast growth factor 21	2.41	1.158e-6
FN1*	Fibronectin 1	2.45	1.174e-5
FZD4	Frizzled Class Receptor 4	-1.52	0.047
GZMA	Granzyme A	-1.78	0.009
GDF3	Growth differentiation factor 3	-1.57	0.024
GREM1	Gremlin1	-1.65	0.022
IL8*	Interleukin 8	-1.67	1.492e-5
KLF4*	Kruppel-like factor 4	1.91	7.551e-6
LCN2*	Lipocalin-2	1.76	1.788e-6
MTDH	Metadherin	-2.21	0.019
OLR-1(LOX-1)*	Oxidized low density lipoprotein receptor 1	2.11	8.457e-6
OPG/TNFRSF11B*	Osteoprotegerin	3.94	2.231e-6
OX40/TNFRSF4	TNF Receptor Superfamily Member 4	-1.45	0.019
PDGFRA*	Platelet-derived growth factor receptor alpha	-1.91	4.375e-6
PDGFRB*	Platelet-derived growth factor receptor beta	-1.48	3.295e-5
PF4*	Platelet factor 4	2.95	8.013e-6
PLG*	Plasminogen	-2.14	7.036e-6
PSEN2	Presenilin 2	-2.43	0.024
SAA1*	Serum Amyloid A1	8.28	9.492e-6
SERTAD2	ERTA Domain Containing 2	-2.61	0.009
TGFBR3*	Transforming growth factor beta receptor 3	-1.71	5.841e-5

TRAIL/TNFSF10*	Tumor Necrosis Factor Superfamily Member 10	-1.68	4.010e-6
VEGFB*	Vascular endothelial growth factor B	-1.71	3.789e-6
VTN*	Vitronectin	-2.03	6.329e-6

* means selective 26 proteins based aortic dissection-related pathway. *p* values are determined by two-tailed Student's *t*-test and are adjusted for multiple comparisons using Benjamin and Hochberg correction.

Supplementary Table 3. Cox proportional hazard analysis of candidate biomarkers for predicting 30-day and post-30-day death in internal and external datasets of derivation cohort.

		Internal Dataset (n=300)				External Dataset (n=236)			
		Uni-variable		Multi-variable		Uni-variable		Multi-variable	
Proteins	Death	HR (95%CI)	*p*-value	HR (95%CI)	*p*-value	HR (95%CI)	*p*-value	HR (95%CI)	*p*-value
OPG/TRAIL	30-day	2.27(1.54-3.33)	3.300e-5	2.05(1.35-3.12)	0.001	2.46(1.70-3.57)	2.000e-6	2.33(1.42-3.82)	0.001
	Post-30-day	2.42(1.48-3.96)	4.350e-4	2.07(1.25-3.43)	0.005	4.56(2.27-9.16)	2.100e-5	4.68(1.72-12.69)	0.002
OPG	30-day	1.67(1.14-2.44)	0.008	1.44(0.96-2.17)	0.076	1.82(1.28-2.60)	0.001	1.44(0.92-2.24)	0.110
	Post-30-day	2.03(1.23-3.34)	0.006	1.98(1.15-3.41)	0.014	2.13(1.18-3.87)	0.013	2.73(1.07-6.95)	0.036
TRAIL	30-day	0.54(0.38-0.77)	0.001	0.49(0.32-0.75)	0.001	0.54(0.38-0.78)	0.001	0.49(0.30-0.79)	0.004
	Post-30-day	0.55(0.35-0.85)	0.007	0.49(0.28-0.83)	0.009	0.51(0.29-0.89)	0.019	0.51(0.22-1.20)	0.123
D-dimer	30-day	1.54(1.07-2.22)	0.020	1.54(1.03-2.30)	0.037	1.54(1.09-2.19)	0.015	1.73(1.11-2.70)	0.016
	Post-30-day	0.94(0.59-1.48)	0.776	0.92(0.54-1.54)	0.738	0.69(0.38-1.25)	0.220	0.49(0.21-1.12)	0.091
SAA1	30-day	1.31(0.92-1.85)	0.133	1.34(0.91-1.95)	0.137	1.18(0.80-1.75)	0.411	1.34(0.81-2.21)	0.254
	Post-30-day	1.10(0.72-1.24)	0.648	1.11(0.71-1.75)	0.649	1.53(0.82-2.86)	0.186	2.48(0.94-6.53)	0.065
LCN2	30-day	1.21(0.86-1.71)	0.295	1.29(0.88-1.89)	0.199	1.31(0.91-1.88)	0.147	1.36(0.86-2.16)	0.186
	Post-30-day	1.19(0.77-1.83)	0.438	1.06(0.67-1.68)	0.804	0.79(0.44-1.44)	0.445	0.97(0.45-2.09)	0.928
FN1	30-day	1.43(1.00-2.04)	0.051	1.34(0.92-1.96)	0.125	1.04(0.72-1.52)	0.831	0.87(0.55-1.38)	0.566
	Post-30-day	0.86(0.55-1.35)	0.511	1.01(0.62-1.67)	0.963	0.91(0.49-1.67)	0.756	1.18(0.50-2.79)	0.705
PF4	30-day	0.72(0.48-1.06)	0.091	0.71(0.46-1.09)	0.119	0.98(0.68-1.41)	0.893	0.88(0.56-1.38)	0.576
	Post-30-day	0.85(0.51-1.40)	0.513	0.83(0.48-1.44)	0.509	1.03(0.59-1.84)	0.924	1.64(0.70-3.82)	0.256
ANGPT1	30-day	0.79(0.54-1.15)	0.216	0.82(0.54-1.23)	0.333	0.99(0.68-1.44)	0.942	1.14(0.71-1.81)	0.590
	Post-30-day	0.64(0.40-1.02)	0.058	0.61(0.37-1.00)	0.050	0.66(0.37-1.20)	0.174	0.76(0.33-1.78)	0.527
PLG	30-day	0.71(0.48-1.04)	0.076	0.73(0.48-1.12)	0.153	0.73(0.49-1.09)	0.121	0.68(0.43-1.10)	0.115
	Post-30-day	0.80(0.49-1.29)	0.357	0.79(0.47-1.33)	0.378	0.73(0.39-1.38)	0.335	0.64(0.27-1.56)	0.329
LOX1	30-day	0.71(0.49-1.00)	0.071	0.82(0.53-1.25)	0.348	0.83(0.58-1.19)	0.305	0.93(0.59-1.47)	0.762

Post-30-day	0.76(0.47-1.24)	0.273	0.89(0.51-1.55)	0.672	1.08(0.60-1.94)	0.794	0.88(0.41-1.87)	0.729

HR with 95%CI associated with 1-SD increase in nine proteins levels, D-dimer and OPG/TRAIL ratio were listed. For adjustment, multivariable included known risk factors (age≥70yrs, SBP, smoking) and previously reported predictors (pain, malperfusion, shock or hypotension, coma or stroke), imaging indicators (thrombosis in false lumen and segment) and surgical treatment. *p* values reported are two-tailed from COX proportional hazard regression analyses.

Supplementary Table 4. C-index of candidate biomarkers for discriminating 30-day/post-30-day death in internal and external datasets of derivation cohort.

Proteins	Death	Internal cohort (n=300)	*p* value	External cohort (n=236)	*p* value
OPG/TRAIL	30-day	0.72(0.64-0.81)	2.367e-7	0.75(0.68-0.83)	2.367e-7
	Post-30-day	0.72(0.61-0.83)	7.537e-5	0.82(0.71-0.92)	7.537e-5
OPG	30-day	0.65(0.56-0.74)	0.001	0.66(0.57-0.75)	0.001
	Post-30-day	0.68(0.56-0.81)	0.003	0.69(0.54-0.84)	0.011
TRAIL	30-day	0.70(0.61-0.78)	6.555e-6	0.66(0.59-0.73)	6.555e-6
	Post-30-day	0.69(0.58-0.80)	0.001	0.75(0.67-0.83)	5.830e-4
D-dimer	30-day	0.60(0.49-0.70)	0.071	0.60(0.51-0.68)	0.031
	Post-30-day	0.52(0.38-0.65)	0.821	0.62(0.48-0.77)	0.090
SAA1	30-day	0.56(0.47-0.64)	0.188	0.54(0.44-0.64)	0.382
	Post-30-day	0.51(0.40-0.61)	0.874	0.61(0.45-0.77)	0.175
LCN2	30-day	0.54(0.45-0.63)	0.404	0.58(0.48-0.68)	0.123
	Post-30-day	0.54(0.42-0.66)	0.515	0.57(0.41-0.73)	0.395
FN1	30-day	0.59(0.49-0.69)	0.093	0.56(0.45-0.66)	0.279
	Post-30-day	0.55(0.42-0.67)	0.450	0.58(0.44-0.72)	0.236
PF4	30-day	0.54(0.44-0.63)	0.430	0.54(0.44-0.64)	0.403
	Post-30-day	0.57(0.47-0.67)	0.168	0.52(0.38-0.66)	0.824
ANGPT1	30-day	0.55(0.44-0.65)	0.379	0.51(0.41-0.61)	0.838
	Post-30-day	0.59(0.48-0.69)	0.095	0.62(0.46-0.78)	0.147
PLG	30-day	0.59(0.51-0.68)	0.032	0.55(0.46-0.65)	0.256
	Post-30-day	0.52(0.42-0.62)	0.748	0.56(0.42-0.71)	0.369
LOX1	30-day	0.56(0.45-0.67)	0.267	0.58(0.50-0.66)	0.042
	Post-30-day	0.57(0.43-0.70)	0.334	0.47(0.40-0.66)	0.642

p values are two-tailed from C-index analyses.

Supplementary Table 5. C-index and three-categories NRI of OPG/TRAIL ratio for predicting the risk of 30-day and post-30-day death in derivation and validation cohorts.

	Derivation Cohorts			Validation Cohort		
	C-index (95%CI)	△C-index (95%CI)	NRI (95%CI)	C-index (95%CI)	△C-index (95%CI)	NRI (95%CI)
30-day Death						
O/T ratio	0.74(0.68-0.79)			0.75(0.70-0.81)		
AAD score	0.58(0.49-0.68)			0.54(0.47-0.62)		
AAD score +O/T ratio	0.74(0.69-0.80)	0.16 (0.06-0.26)	0.18(0.14-0.21) Events: 0.08(0.18-0.15) No-events: 0.09(0.07-0.12)	0.75(0.70-0.81)	0.21 (0.12-0.30)	0.51(0.46-0.56) Events: 0.38(0.27-0.49) No-events: 0.13(0.09-0.17)
Post-30-day Death						
O/T ratio	0.76(0.68-0.84)			0.84(0.72-0.97)		
Clinical Predictors	0.63(0.54-0.71)			0.81(0.74-0.89)		
Clinical Predictors +O/T ratio	0.78(0.71-0.85)	0.10 (0.12-0.19)	0.44(0.40-0.49) Events: 0.16(0.04-0.29) No-events: 0.28(0.24-0.32)	0.89(0.79-0.99)	0.07 (0.04-0.11)	0.49(0.43-0.54) Events: 0.42(0.09-0.74) No-events: 0.07(0.04-0.10)

Clinical predictors included stroke, chronic renal dysfunction, myocardial infarction, older age, atherosclerosis history, previous cardiac surgery and female. O/T=OPG/TRAIL.

Supplementary Table 6. Two-categories NRI for OPG/TRAIL ratio over AAD score or clinical predictors in derivation and validation cohorts.

	Derivation Cohort			Validation Cohort		
	Over-all death NRI (95%CI)	30-day death NRI (95%CI)	Post-30-day death NRI (95%CI)	Over-all death NRI (95%CI)	30-day death NRI (95%CI)	Post-30-day death NRI (95%CI)
AAD score +O/T ratio	0.53(0.49-0.57) Events: 0.21(0.13-0.29) No-events: 0.32(0.28-0.37)	-0.01(-0.02-0.00) Events: 0.13(0.05-0.20) No-events: -0.14(-0.17--0.11)		0.64(0.60-0.69) Events: 0.31(0.21-0.41) No-events: 0.34(0.28-0.39)	0.34(0.29-0.38) Events: 0.33(0.22-0.44) No-events: 0.01(0.00-0.02)	
Clinical Predictors +O/T ratio	0.17(0.14-0.21) Events: 0.09(0.04-0.15) No-events: 0.08(0.06-0.11)		0.05(0.03-0.07) Events: 0.09(0.00-0.18) No-events: -0.04(-0.05--0.02)	0.29(0.25-0.34) Events: 0.17(0.09-0.25) No-events: 0.12(0.09-0.16)		0.24(0.19-0.29) Events: 0.25(-0.04-0.54) No-events: -0.01(-0.02-0.00)

Clinical predictors included stroke, chronic renal dysfunction, myocardial infarction, older age, female, atherosclerosis history and previous cardiac surgery. O/T=OPG/TRAIL.

Supplementary Table 7. Performance of OPG/TRAIL ratio-based risk stratification for predicting the risk of 30-day and post-30-day death in derivation and validation cohorts

Cohort	Patient (%)	Risk Stratification	NPV	PPV	Sensitivity	Specificity
30-day Death						
Derivation	95(17.7%)	Low risk	96.8%	15.7%	95.8%	19.9%
	63(11.8%)	High risk	90.1%	39.7%	34.7%	91.8%
Validation	78(19.5%)	Low risk	97.4%	23.0%	97.4%	23.5%
	44(11.0%)	High risk	86.2%	61.4%	35.5%	94.8%
Post-30-day Death						
Derivation	91(20.0%)	Low risk	96.7%	9.32%	91.9%	21.0%
	38(8.3%)	High risk	93.8%	29.0%	29.7%	93.6%
Validation	76(23.5%)	Low risk	98.7%	4.4%	91.7%	24.0%
	17(5.25%)	High risk	97.4%	23.5%	33.3%	95.8%

NPV, PPV, sensitivity and specificity were calculated to estimate the performance of OPG/TRAIL ratio-based risk stratification for 30-day/post-30-day death. NPV: negative predictive value; PPV: positive predictive value.

Supplementary Table 8. Sensitivity analysis of hazard ratios for OPG/TRAIL ratio for death in surgical patients.

	Events/ All patients	Crude HR (95% CI)	*p* value	Adjusted HR (95%CI)	*p* value
Derivation Cohort					
Over-all death	70/462	2.58 (1.95-3.42)	3.678e-11	2.51 (1.86-3.38)	1.364e-09
30-day death	44/462	2.36 (1.67-3.35)	1.000e-06	2.32 (1.61-3.36)	7.000e-06
Post-30-day death	26/435	3.02 (1.88-4.86)	5.000e-06	2.85 (1.73-4.72)	4.400e-05
Validation Cohort					
Over-all death	67/363	3.05 (2.22-4.19)	5.120e-12	3.25 (2.13-4.96)	4.770e-08
30-day death	57/363	2.83 (2.02-3.96)	1.507e-09	2.84 (1.82-4.43)	4.000e-06
Post-30-day death	10/306	5.00 (2.01-12.43)	0.001	8.27 (2.19-31.27)	0.002

HR with 95%CI associated with 1-SD increase of OPG/TRAIL ratio were listed. For adjustment, variables included known risk factors (age≥70yrs, SBP, smoking) and previously reported predictors (pain, malperfusion, shock or hypotension, coma or stroke), and imaging indicators (thrombosis in false lumen and segments). *p* values reported are two-tailed from COX proportional hazard regression analyses.

Supplementary Table 9. Causes of death in the derivation and validation cohorts.

	Internal dataset		External Dataset		Validation cohort	
	30-day	Post-30-day	30-day	Post-30-day	30-day	Post-30-day
Total	36	23	36	14	76	12
Multiple organ dysfunction syndrome	11(30.6)	10(43.5)	12(33.3)	4(28.6)	20(26.3)	3(25.0)
Septicemia	2(5.6)	0	1(2.8)	0	2(2.6)	1(8.3)
Renal failure	2(5.6)	1(4.3)	1(2.8)	0	6(7.9)	1(8.3)
Heart failure	1(2.8)	2(8.7)	4(11.1)	2(14.3)	3(3.9)	1(8.3)
Acute respiratory distress syndrome	2(5.6)	1(4.3)	2(5.6)	1(7.1)	3(3.9)	2(16.7)
Rupture	13(36.1)	3(13.0)	13(36.1)	4(28.6)	36(47.4)	2(16.7)
Cerebral hemorrhage or infarction	5(13.9)	4(17.4)	3(8.3)	2(14.3)	5(6.6)	3(25.0)
Uncertain	0	2(8.7)	0	1(7.1)	1(1.3)	0

Supplementary Figure 1. Screening of TA-AAD associated proteins.

a. Selection strategy used to identify 26 candidate proteins. Selection used a summarized metric to find the proteins most related to aortic dissection-related pathological processes. **b.** Heat map of the 26 proteins whose serum levels significantly differed in discovery cohort 2, including 31 TA-AAD patients and 32 health controls. The *p*-values presented have been adjusted for multiple comparisons (number of proteins) by using Benjamin and Hochberg correction. *p* values are determined by two-tailed Student's *t*-test and are adjusted for multiple comparisons using Benjamin and Hochberg correction. **c-o.** The serum concentration of 13 candidate proteins in screening set 3 including 77 TA-AAD patients and 200 healthy controls. The serum CNTF was not detected by ELISA. Box plots indicate median (middle line), 25th, 75th percentile (box) and 5th and 95th percentile (whiskers) as well as outliers (single points). *p* values were two-tailed from Mann-Whitney *U* tests. SMC: Smooth muscle cell; ECM: extracellular matrix.

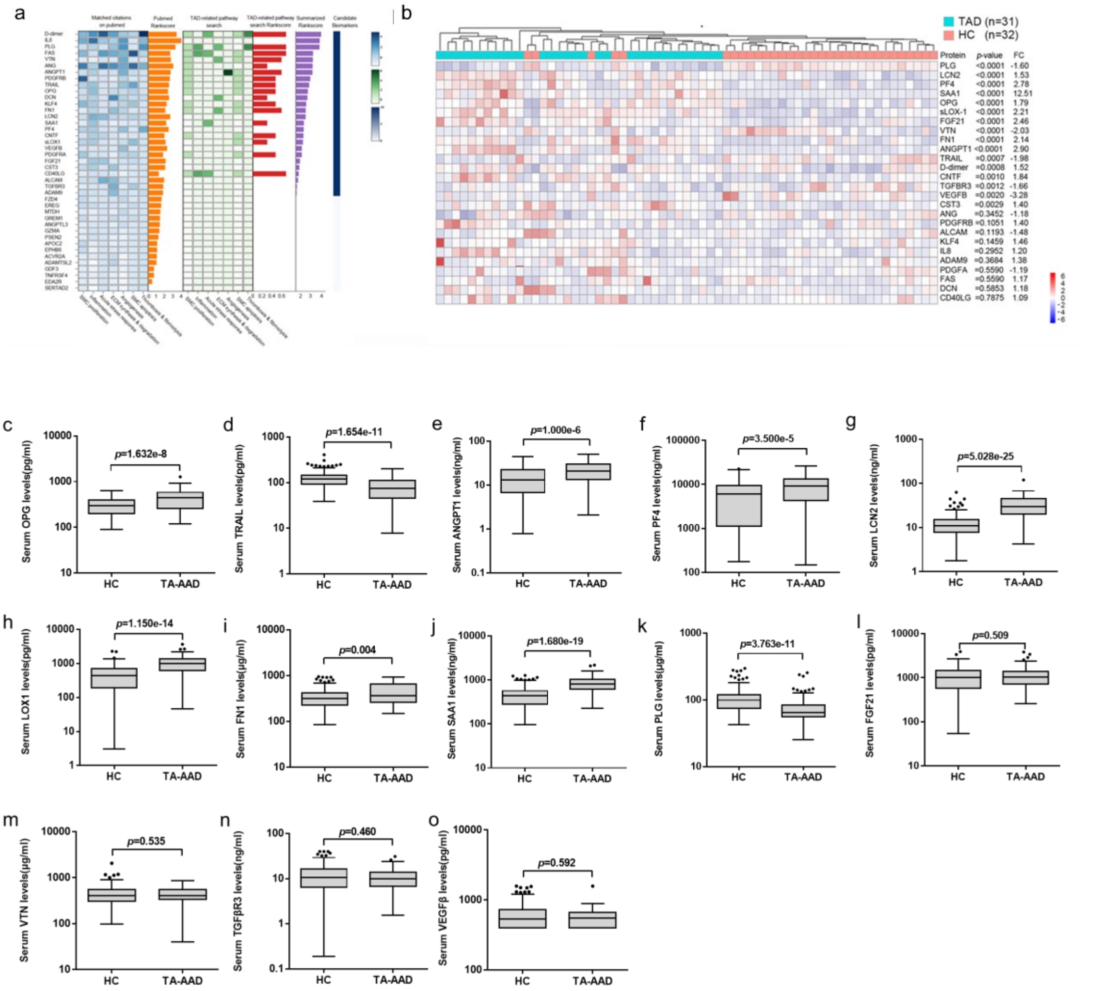

Supplementary Figure 2. The serum concentrations of 9 candidate proteins in serum of patients in the internal and external datasets of derivation cohort. **a-j.** Concentrations of 9 proteins (LCN2, SAA1, sLOX-1, PF4, TRAIL, OPG, ANGPT1, PLG, and FN1) and OPG/TRAIL ratio in the patients who lived and the patients who died in the internal cohort (n=241 vs. n=59) and external cohort (n=186 vs. n=50). Boldface horizontal lines are means. Box plots indicate median (middle line), 25th, 75th percentile (box) and 5th and 95th percentile (whiskers) as well as outliers (single points). *p* values are two-tailed from Mann-Whitney *U* tests. TA-AAD=Type A acute aortic dissection.

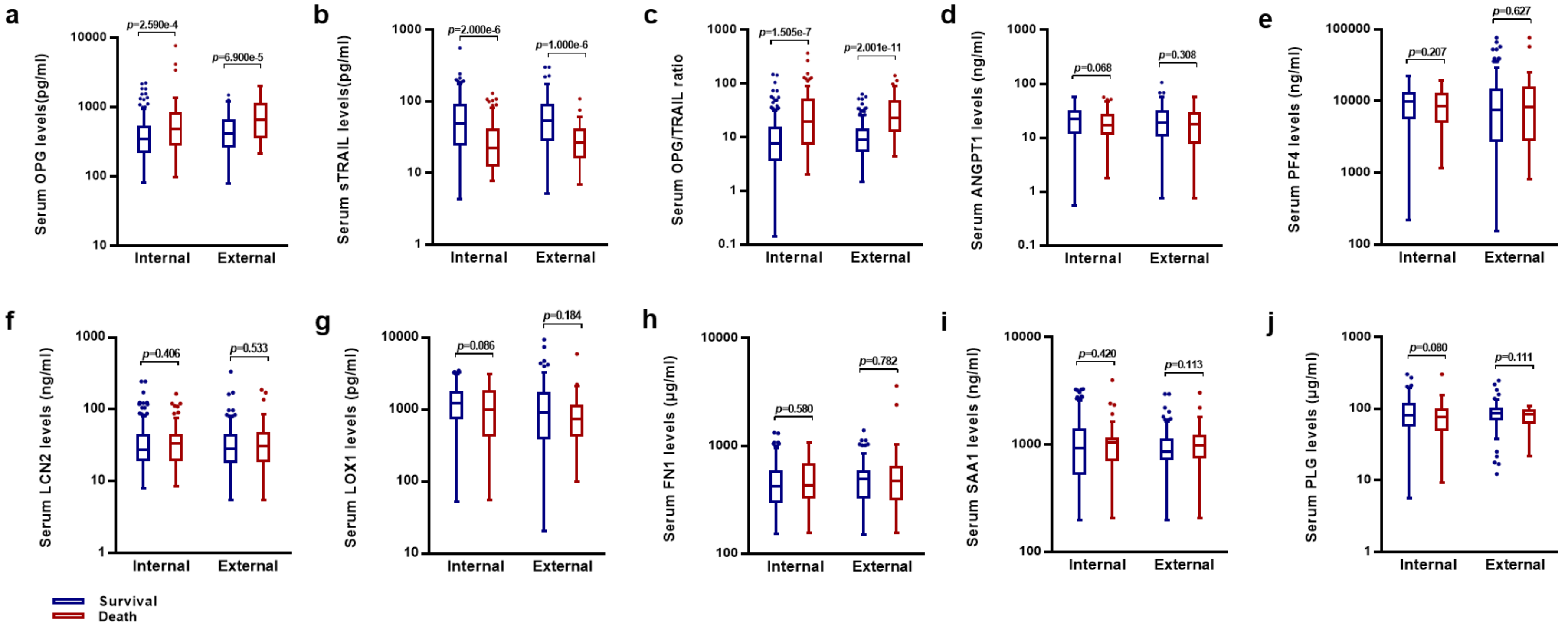

Supplementary Figure 3. Three-categories NRI of OPG/TRAIL ratio for over-all death on the existing models in derivation and validation cohorts.

Patients were divided into subgroups that did or did not reach the primary endpoint of over-all death. The number (percentage) of patients in each risk category was shown. O/T ratio= OPG/TRAIL ratio. NRI: net reclassification improvement.

a. Derivation Cohort: vs. AAD score

	**AAD score +O/T ratio**			
	**<5%**	**5-20%**	**>20%**	**All**
**Patients without events**				
AAD score				
<5%	1(0.2)	0	0	1(0.2)
5-20%	23(5.4)	116(27.2)	35(8.2)	174(40.7)
>20%	18(4.2)	154(36.1)	80(18.7)	252(59.0)
All	42(9.8)	270(63.2)	115(26.9)	427(100)
				NRIne=0.374
**Patients with events**				
AAD score				
<5%	16(14.7)	8(7.3)	2(1.8)	26(23.9)
5-20%	11(10.1)	41(37.6)	25(22.9)	77(70.6)
>20%	1(0.9)	3(2.8)	2(1.8)	6(5.5)
All	28(25.7)	52(47.7)	29(26.6)	109(100)
				NRIe=0.183

b. Derivation Cohort: vs. Clinical predictors

	**Clinical Predictors +O/T ratio**			
	**<5%**	**5-20%**	**>20%**	**All**
**Patients without events**				
Clinical Predictors				
<5%	3(0.7)	1(0.2)	0	4(0.9)
5-20%	41(9.6)	181(42.4)	52(12.2)	274(64.2)
>20%	0	87(20.4)	62(14.5)	149(34.9)
All	44(10.3)	269(63.0)	114(26.7)	427(100)
				NRIne=0.186
**Patients with events**				
Clinical Predictors				
<5%	14(12.8)	8(7.3)	0	22(20.2)
5-20%	12(11.0)	38(34.9)	18(16.5)	68(62.4)
>20%	0	8(7.3)	11(10.1)	19(17.4)
All	26(12.9)	54(49.5)	29(26.6)	109(100)
				NRIe=0.055

c. Validation Cohort: vs. AAD score

	**AAD score +O/T ratio**			
	**<5%**	**5-20%**	**>20%**	**All**
**Patients without events**				
AAD score				
<5%	0	0	0	0
5-20%	22(7.1)	64(20.5)	24(7.7)	110(35/3)
>20%	20(6.4)	109(34.9)	73(23.4)	202(64.7)
All	42(13.5)	173(55.4)	97(31.1)	312(100)
				NRIne=0.407
**Patients with events**				
AAD score				
<5%	11(12.5)	10(11.4)	0	21(23.9)
5-20%	9(10.2)	20(22.7)	29(33.0)	58(65.9)
>20%	0	2(2.3)	7(8.0)	9(10.2)
All	20(22.7)	32(36.4)	36(40.9)	88(100)
				NRIe=0.318

d. Validation Cohort: vs. Clinical predictors

	**Clinical Predictors +O/T ratio**			
	**<5%**	**5-20%**	**>20%**	**All**
**Patients without events**				
Clinical Predictors				
<5%	2(0.6)	0	0	2(0.6)
5-20%	58(18.6)	90(28.8)	34(10.9)	182(58.3)
>20%	15(4.8)	57(18.3)	56(17.9)	128(41.0)
All	75(24.0)	147(47.1)	90(28.8)	312(100)
				NRIne=0.308
**Patients with events**				
Clinical Predictors				
<5%	15(17.0)	5(5.7)	1(1.1)	21(23.9)
5-20%	3(3.4)	24(27.3)	17(19.3)	44(50.0)
>20%	0	3(3.4)	20(22.7)	23(26.1)
All	18(20.5)	32(36.4)	38(43.2)	88(100)
				NRIe=0.193

Supplementary Figure 4. Size of low-risk and high-risk subpopulation of patients as a function of risk threshold of OPG/TRAIL ratio for over-all death in derivation cohort.

A series of NPVs or PPVs were listed to find most suitable values as the threshold to classify patients as low risk (a) or high risk (b), respectively. Vertical dashed lines indicated the selected NPV/PPV and what it corresponds to. NPV: negative predictive value; PPV: positive predictive value.

a. Low-risk subpopulation of patients

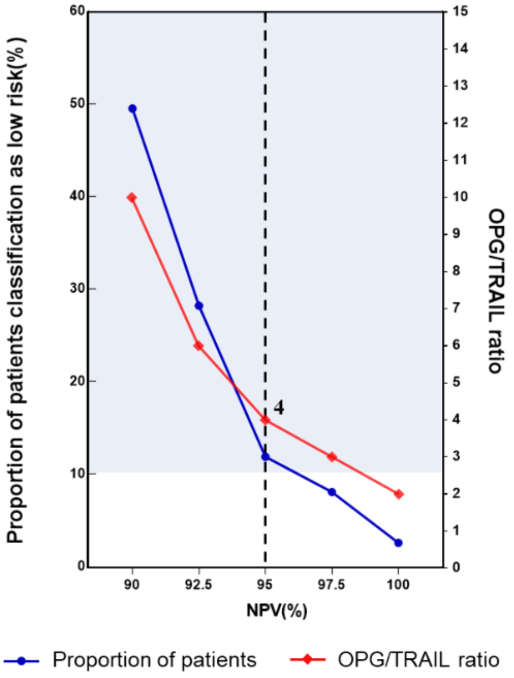

b. High-risk subpopulation of patients

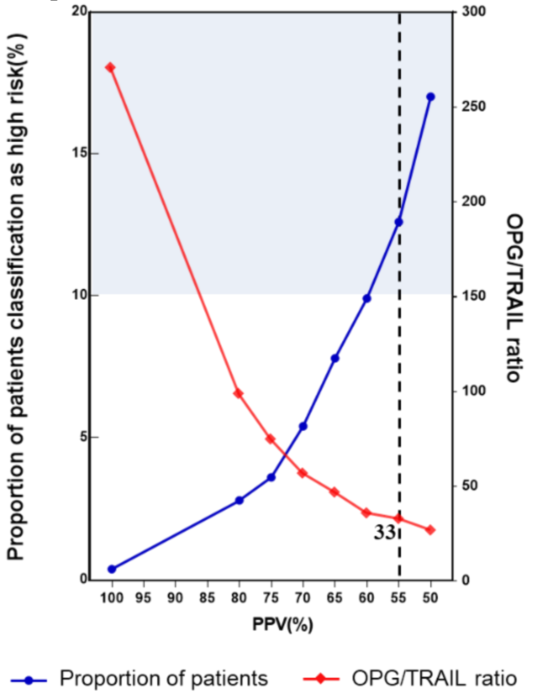

Supplementary Figure 5. Multivariable-adjusted restricted cubic spline analyses in derivation and validation cohorts

Possible nonlinear relationships between OPG/TRAIL ratio and over-all death were examined with restricted cubic splines in derivation cohort (n=536) (a) and validation cohort (n=400) (b). Adjusted for known risk factors (age≥70yrs, SBP, smoking), previously reported predictors (pain, malperfusion, shock or hypotension, coma or stroke), imaging indicators (thrombosis in false lumen and segment), and surgical treatment.

a. Derivation cohorts

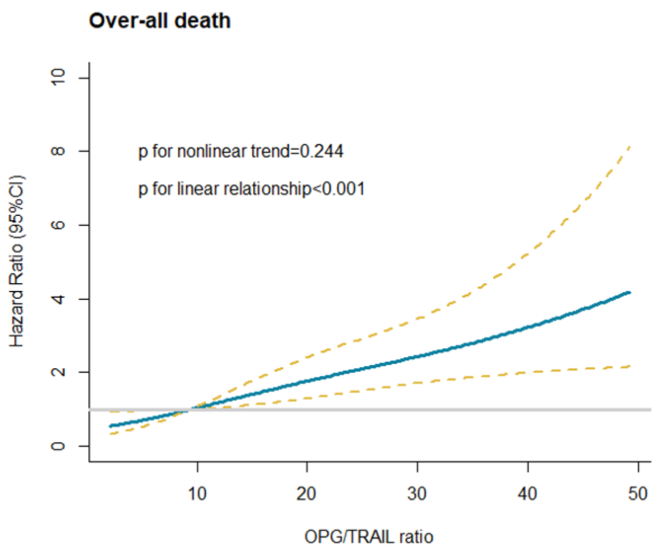

b. Validation cohort

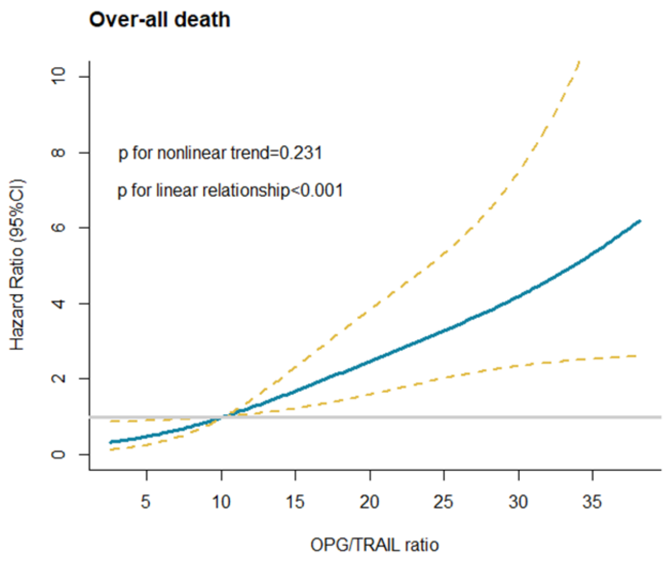

Supplementary Figure 6. Subgroups analysis of hazard ratios for OPG/TRAIL ratio for over-all death in derivation and validation cohorts.

Subgroups used were sex (male vs. female), age (⩾60 vs.<60), hypertension history, smoking history and diabetes mellitus history. HRs with 95% CIs associated with 1-SD increase in OPG/TRAIL ratio levels were evaluated in the derivation cohort (n=536) and validation cohort (n=400). Boxes represent HR. Error bars represent 95% CI. *p* values reported are two-tailed from COX proportional hazard regression analyses. HR=hazard ratio. DM: diabetes mellitus.

a. Derivation Cohorts

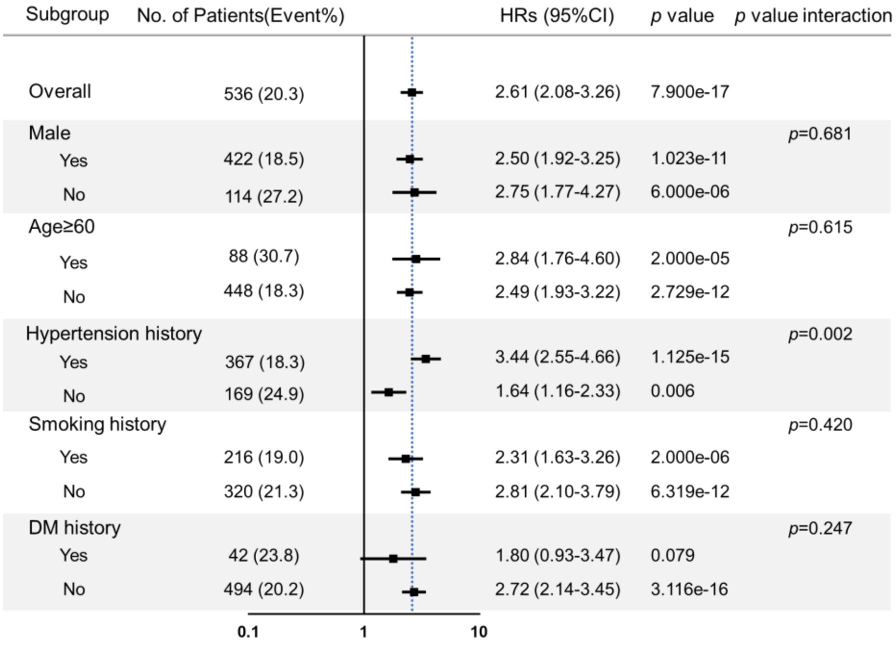

b. Validation Cohort

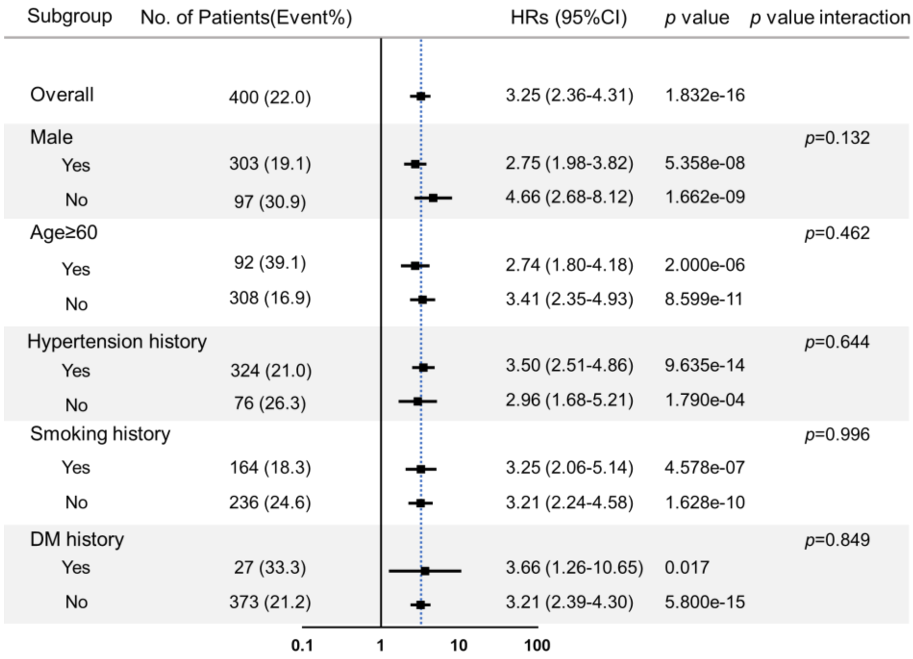

Supplementary Figure 7. Formation of the screening and an internal dataset of derivation from patients hospitalized with TA-AAD.

The patients enrolled from Beijing Anzhen Hospital. After excluding 76 patients for the reasons shown, a total of 420 patients was divided into the screening and an internal cohort. TAD=thoracic aortic dissection. TA-AAD=Type A acute aortic dissection.

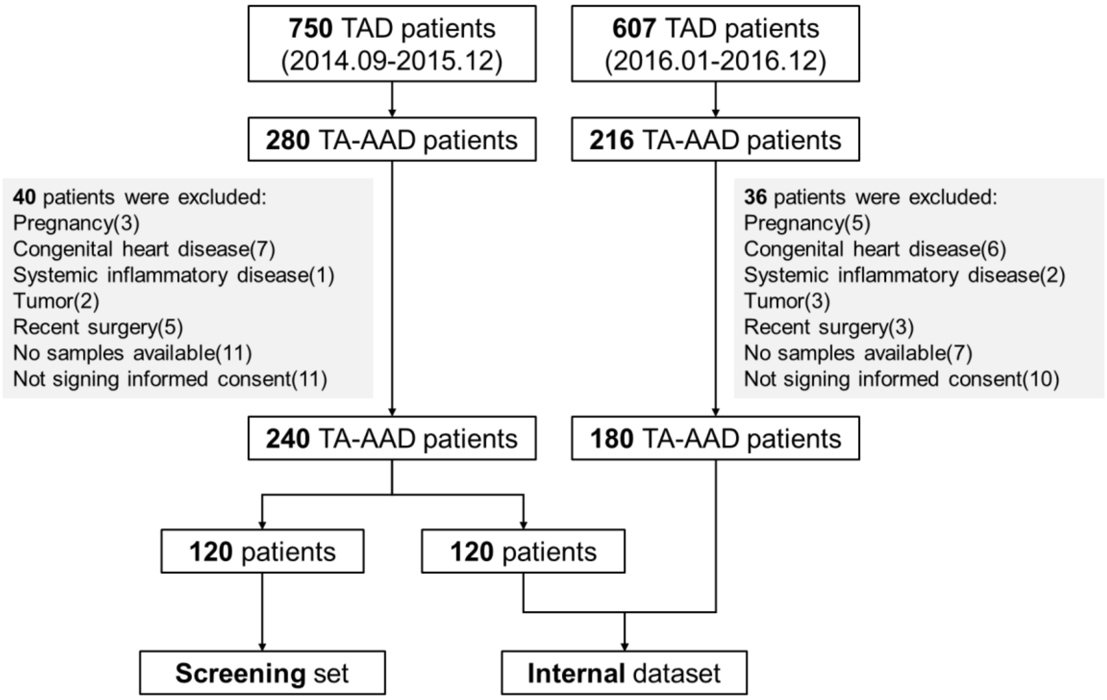

Supplementary Figure 8. A copy of the Dara Access Agreement.

The Beijing Anzhen Hospital (AZH) of Capital Medical University and the Recipient hereby enter into this Agreement for the transfer of clinical data used in the paper by L.J, et al.

In consideration of AZH providing data to Recipient, Recipient agrees to the following terms and conditions:

1. Data will be provided to Recipient with a Research Plan that is approved by AZH.
2. Data will be used only by Recipient for purpose described in the Research Plan.
3. The Recipient will not release data to a third party without prior approval from AZH.
4. The Recipient will not share, publish, or otherwise release any findings or conclusions derives from analysis of data obtained from AZH without prior approval form AZH.
5. All data transferred to Recipient shall remain the property of AZH.

____

For the Beijing Anzhen Hospital of Capital Medical University

Date: _____________________________

____

For the Recipient

Date: _____________________________
