# Supplementary material for: OPG/TRAIL ratio as a predictive biomarker of mortality in patients with type A acute aortic dissection
Source: Nat Commun. 2021 Jun 7;12:3401. doi: 10.1038/s41467-021-23787-5 (PMC8185077; doi:10.1038/s41467-021-23787-5)
Supplement: Supplementary file 3 — Description of Additional Supplementary Files [file 41467_2021_23787_MOESM3_ESM.docx]

Description of Additional Supplementary File

File Name: Supplementary Data 1
Description: The commercialised antibody array containing 1000 proteins between 12 patients with TA-AAD and 12 healthy controls in the screening set 1.
