# Supplementary material for: OPG/TRAIL ratio as a predictive biomarker of mortality in patients with type A acute aortic dissection
Source: Nat Commun. 2021 Jun 7;12:3401. doi: 10.1038/s41467-021-23787-5 (PMC8185077; doi:10.1038/s41467-021-23787-5)
Supplement: Supplementary file 5 — Reporting Summary [file 41467_2021_23787_MOESM5_ESM.pdf]

nature research

Corresponding author(s): Yulin Li; Jie Du

Last updated by author(s): Mar 24, 2021

Reporting Summary

Nature Research wishes to improve the reproducibility of the work that we publish. This form provides structure for consistency and transparency in reporting. For further information on Nature Research policies, see our Editorial Policies and the Editorial Policy Checklist.

Statistics

For all statistical analyses, confirm that the following items are present in the figure legend, table legend, main text, or Methods section.

n/a	Confirmed	
☐	☒	The exact sample size (*n*) for each experimental group/condition, given as a discrete number and unit of measurement
☐	☒	A statement on whether measurements were taken from distinct samples or whether the same sample was measured repeatedly
☐	☒	The statistical test(s) used AND whether they are one- or two-sided<br>*Only common tests should be described solely by name; describe more complex techniques in the Methods section.*
☐	☒	A description of all covariates tested
☐	☒	A description of any assumptions or corrections, such as tests of normality and adjustment for multiple comparisons
☐	☒	A full description of the statistical parameters including central tendency (e.g. means) or other basic estimates (e.g. regression coefficient) AND variation (e.g. standard deviation) or associated estimates of uncertainty (e.g. confidence intervals)
☐	☒	For null hypothesis testing, the test statistic (e.g. *F*, *t*, *r*) with confidence intervals, effect sizes, degrees of freedom and *P* value noted<br>*Give P values as exact values whenever suitable.*
☒	☐	For Bayesian analysis, information on the choice of priors and Markov chain Monte Carlo settings
☐	☒	For hierarchical and complex designs, identification of the appropriate level for tests and full reporting of outcomes
☐	☒	Estimates of effect sizes (e.g. Cohen's *d*, Pearson's *r*), indicating how they were calculated

Our web collection on statistics for biologists contains articles on many of the points above.

Software and code

Policy information about availability of computer code

Data collection	No software was used for data collection.
Data analysis	SPSS version 23.0 (IBM Corp.) and R version 3.3.3 (R Foundation for Statistical Computing, Vienna, Austria) were used for data analyses. We used the rms package(version 6.2-0), survival package(version 3.2-10), and survcomp package(version 1.30.0) in R programming. The R code used are available from the corresponding author upon request.

For manuscripts utilizing custom algorithms or software that are central to the research but not yet described in published literature, software must be made available to editors and reviewers. We strongly encourage code deposition in a community repository (e.g. GitHub). See the Nature Research guidelines for submitting code & software for further information.

Data

Policy information about availability of data

All manuscripts must include a data availability statement. This statement should provide the following information, where applicable:

- Accession codes, unique identifiers, or web links for publicly available datasets
- A list of figures that have associated raw data
- A description of any restrictions on data availability

The data supporting the findings in this study are available in the Supplementary Information or from the corresponding author upon reasonable request.

Field-specific reporting

Please select the one below that is the best fit for your research. If you are not sure, read the appropriate sections before making your selection.

- [x] Life sciences
- [ ] Behavioural & social sciences
- [ ] Ecological, evolutionary & environmental sciences

For a reference copy of the document with all sections, see nature.com/documents/nr-reporting-summary-flat.pdf

Life sciences study design

All studies must disclose on these points even when the disclosure is negative.

Sample size	Calculation of sample size were considered in different stages. In derivation cohort, to achieve 90% power at 5% significance with a 4:1 sampling ratio (because 25% of reported mortality rate), we required 504 participants to detect an overall survival hazard ratio of 1.5. In validation cohort, the sample size estimation based on preliminary data of derivation cohort. Assuming a mortality rate of 20%, a sample size of 187 patients would be powered (90% at p<0.05) to detect a HR of OPG/TRAIL ratio of 2.0.
Data exclusions	No data is excluded from the analysis.
Replication	The adverse prognostic events in patients with TA-AAD were assessed independently by two experienced vascular specialists. All attempts of replication were successful.
Randomization	The participants in this study were recruited consecutively.
Blinding	The technicians and statisticians have no knowledge of each other's content and the determination of outcome events.

Reporting for specific materials, systems and methods

We require information from authors about some types of materials, experimental systems and methods used in many studies. Here, indicate whether each material, system or method listed is relevant to your study. If you are not sure if a list item applies to your research, read the appropriate section before selecting a response.

Materials & experimental systems

n/a	Involved in the study	
x		Antibodies
x		Eukaryotic cell lines
x		Palaeontology and archaeology
x		Animals and other organisms
	x	Human research participants
	x	Clinical data
x		Dual use research of concern

Methods

n/a	Involved in the study	
x		ChIP-seq
x		Flow cytometry
x		MRI-based neuroimaging

Human research participants

Policy information about studies involving human research participants

Population characteristics	Patient characteristics are detailed in Table 1.
Recruitment	Patients were continuously recruited from the Beijing Anzhen Hospital, the First Affiliated Hospital of Sun Yat-sen Univerity, the Xi Jing Hospital, the First Affiliated Hospital of Dalian Medical University, and the Tongji Hospital. Healthy subjects were recruited by the Health Examine Centre of the the Beijing Anzhen Hospital. Neither self-selection bias or other biases are present in our study.
Ethics oversight	This study was approved by the ethics committees or institutional review boards of all five medical centers. All participants provided written informed consent. The study design and conduct complied with all relevant regulations regarding the use of human study participants and was conducted in accordance to the Declaration of Helsinki.

Note that full information on the approval of the study protocol must also be provided in the manuscript.

Clinical data

Policy information about clinical studies

All manuscripts should comply with the ICMJEguidelines for publication of clinical research and a completedCONSORT checklist must be included with all submissions.

Clinical trial registration	ClinicalTrials.gov Identifier: NCT03010514"A Registry Study on Genetics and Biomarkers of Thoracic Aortic Aneurysm/Dissection"

Study protocol	The full study protocol can be assessed in the supplementary methods.
Data collection	Data collection for present study started in 2014 and is ongoing. Blood samples and clinical information were collected on the Beijing Anzhen Hospital, the First Affiliated Hospital of Sun Yat-sen Univerity, the Xi Jing Hospital, the First Affiliated Hospital of Dalian Medical University, and the Tongji Hospital. The blood sample was drawn within 48 hours of admission before administration of operative treatment.
Outcomes	The primary endpoint was overall mortality and the secondary endpoint were 30-day and post-30-day mortality.
